# Vaccines for Influenza

**DOI:** 10.3390/vaccines9010047

**Published:** 2021-01-14

**Authors:** Effie-Photini Tsilibary, Spyros A. Charonis, Apostolos P. Georgopoulos

**Affiliations:** 1 Brain Sciences Center, Department of Veterans Affairs Health Care System, Minneapolis, MN 55417, USA; tsili001@umn.edu (E.-P.T.); scharoni@umn.edu (S.A.C.); 2 Department of Neuroscience, University of Minnesota Medical School, Minneapolis, MN 55455, USA

Two reviews by Harding and Heaton [1] and Lewnard and Cobey [2] shed light on recent progress and new developments towards a universal influenza vaccine and the effectiveness of the influenza vaccine. The first review [1] discusses the development of a universal influenza vaccine as one of pivotal importance, given that influenza viruses infect approximately 20% of the global population annually and result in hundreds of thousands of deaths. In an attempt to reduce influenza disease burden while universal vaccines are developed and tested, many groups are working on different strategies to improve the efficacy of the standard seasonal vaccine and to more accurately determine the effectiveness of the different vaccines statistically.

*Types of Influenza Vaccines*

The review by Harding and Heaton [1] hinges on the strategies for most effective vaccines, being in a “trivalent” or “quadrivalent” format, wherein the vaccine is a cocktail of influenza type A (H1N1 and H3N2 viruses) and two major lineages of the B strain. Seasonal vaccines are regularly required due to the “antigenic drift” caused by the high rate of mutations of the influenza RNA (compared to DNA viruses) as a result of the lower fidelity of RNA polymerases. For vaccine development, once a strain is selected, the genetic segments encoding the two major glycoproteins, hemagglutinin (HA) and neuraminidase (NA), of the circulating influenza viruses are used and screened to isolate “candidate vaccine viruses”. The main methods are as follows. (a) In egg-based methods, the generated candidate viruses are grown in embryonated chicken eggs to eventually grow high titers; this approach has been used for decades because it can produce high numbers of vaccines, up to 1.5 billion doses, to meet the annual need of the new seasonal vaccines. However, some H3N2 strains grow poorly in eggs, and, additionally, this approach requires an enormous supply of chicken eggs, sometimes slowing production depending on egg availability. An improvement of this approach is focused on engineering recombinant viruses with desired characteristics for vaccines that can be grown in chicken eggs, avoiding egg-adaptive mutations. A general drawback of egg-based vaccines is that they are not suitable for people with allergy to eggs. (b) In a cell-based influenza vaccine manufacturing platform, Madin-Darby Canine Kidney (MDCK) cells are used, in which reassortant viruses are generated using HA and NA components of influenza strains through the use of baculoviruses, among others. The use of cells reduces the potential constraints of egg shortages and also has the advantage that glycosylation of HA and NA is of the mammalian and not the avian species. The main drawback is that influenza viruses can develop mutations in HA and NA proteins after serial passaging in cell culture. (c) In methods using nanoparticles, HA expressed in a cell line is purified and assembled into a nanoparticle for use in the vaccine, a method still in the preclinical phase. (d) Peptide-based vaccines are based on the synthesis of specific epitopes from influenza proteins, recognized by B- and T-cells. The peptides are loaded on liposomes or virosomes, which target cells of interest to deliver antigen. Finally, (e) nucleic acid vaccines rely on recombinant DNA or RNA molecules. The antigen is cloned into an expression plasmid and propagated in *Escherichia coli* cells. The RNA or the DNA is then purified and often administered via injection. Host cells take up RNA or DNA and begin expressing the desired antigen. The latter approaches have been used in a variety of preclinical and phase 1 clinical trials, typically expressing at least the HA protein of the desired virus, and show a great deal of promise for future clinical trials and subsequent development. Lately, due to the COVID-19 pandemic, this technology has advanced with great speed, and two RNA vaccines for SARS-CoV-2, BNT162b2 mRNA by Pfizer-BioNTech [3] and SARS-CoV-2 mRNA-1273 by Moderna [4], have completed phase 3 clinical trials successfully and are being currently administered. Newer information since this review hinges on more efforts for a better influenza vaccine [5], complying with the 2017 four criteria from the National Institute of Allergy and Infectious Diseases (NIAID) in the US, [5] namely (1) 75% effectiveness against symptomatic influenza infection, (2) protection against both group I and group II influenza viruses, (3) durable protection that lasts at least 1 year, and (4) suitable for all age groups.

Altering glycan composition on recombinant HA and split virus vaccines is one strategy for a universal vaccine, since the addition of N-glycosylation sites on the immunodominant HA head domain allow the virus to use N-glycans to escape host immune system recognition by hiding, using the so-called “glycan shielding”. These glycan regions of influenza hemagglutinin may be used as a target to increase the breadth of protection by the influenza vaccine. To get rid of these “glycan shields”, human embryonic kidney cells that are unable to synthesize complex type N-glycans are used to produce truncated hemagglutinins having only high mannose residues on their N-glycosylation sites [5]. The mannose residues are then trimmed enzymatically to yield a single glycan residue (“monoglycosylation concept”), and thus the size and the shielding effect of N-glycans are dramatically reduced but the native HA structure remains intact, resulting in antibodies with better binding affinity, neutralization, and cross-reactivity as compared to the native HA [5]. This recombinant HA vaccine could be advantageous for speed, flexibility, and safety, but egg-based production, currently the mainstream for influenza vaccine manufacture, shares the limitations of this approach. Initial efforts in this direction have yielded a monoclycosylated H1N1 virus vaccine that offers cross-protection against diverse influenza strains [5].

Other types of vaccines are also being developed based on recombinant HA. For example, Novavax has used the baculovirus expression system to produce recombinant HAs, which form detergent-protein nanoparticles in saponin adjuvant, the latter inducing, in ferrets and in clinical trials phases 1 and 2, higher levels of neutralizing antibodies compared to commercial inactivated vaccines, (trivalent Fluzone) [5]. Some efforts are focused on the more conserved stem region of HA rather than the highly variable head domain to better stimulate the immune system [5]. Other vaccine types use peptide-based epitopes from HA, nucleoprotein (Np), and matrix 1 (M1) protein for B- and T-cells (BiondVax Pharmaceuticals, Jerusalem, Israel) (http://www.biondvax.com/), PepTcell by Seek, London, UK) (https://www.seekacure.com/) [5], some using in silico multiple alignment of influenza sequences to predict epitopes that would be good candidates for vaccine development; these vaccine efforts are in phases 1 and 2 clinical trials with positive results for cellular and humoral immunity. Finally, Codagenix, Farmingdale, NY, USA, uses attenuated influenza vaccine (CodaVax: https://codagenix.com/vaccine-programs/influenza/ [5]) with an approach in which viral proteins have the same amino acid sequence and antigenicity as wild type strains but are attenuated due to excessive use of rare codons [5].

For all these vaccines, vaccine efficiency (VE), discussed in the second review [2], is one of the three major areas of the NIAID’s outlines requiring improvement. The mandate is to meet one of three conditions: seroprotection (HI titer of ≥1:40 or SRH of 25 mm^2^) rate of over 70%, seroconversion (4-fold increase in titer) rate more than 40%, or a geometric mean increase (pre- vs. post-vaccination) of 2.5 times in healthy adults, and 60, 30%, 2.0× respectively, for elders. It appears that cell-based manufacture is the future for influenza vaccines, a plethora of a next generation which is currently under development. WHO expects a universal influenza A vaccine to be in advanced clinical trials as early as 2027, the factors of clinical safety and efficacy remaining a major hurdle towards approval [5].

Quite recently, a new vaccine, the recombinant quadrivalent virus-like particle (QVLP) influenza vaccine containing influenza hemaggutinins expressed with the use of a plant-based platform, was demonstrated to be equally effective as the other quadrivalent vaccines in phase 3 clinical trials and is awaiting approval, allowing for a cheaper and faster influenza vaccine production [6].

*Variability of Efficacy of Influenza Vaccines in Different Studies*

The second review [2] addresses the reasons for the observed variability of effectiveness of different vaccines used, analyzing factors such as variability of the immune response, temporal and demographic patterns underlying estimates of influenza vaccine effectiveness, and limitations of the epidemiological methods used to measure influenza vaccine performance. For vaccine effectiveness, the required information is the reduction a vaccine confers to an individual’s susceptibility to influenza infection or influenza-caused illness compared to the same individual’s susceptibility without the vaccine. This is termed “vaccine efficacy” (in randomized studies) “or vaccine effectiveness” (in observational studies of individual outcomes), both referred to as VE. The problem with epidemiological studies of VE is that these do not rely on randomized trials or observational cohort studies but on the prevalence of prior vaccination among individuals who seek care for influenza-like illness and receive a positive or a negative outcome of a laboratory test for influenza infection, a process called “test negative design” (TND). This method resembles a “retrospective cohort” study questioned for its use in policy making, since its observational nature introduces bias to the point that vaccines that are actually protective can appear to facilitate infection. The reason is that individual decisions to vaccinate may be associated with other factors influencing their exposure or susceptibility to influenza. An ideal study should stratify VE according to past vaccination, exposure, and infection history in order to isolate the effect of vaccination on susceptibility to infection within the same season. The all-or-nothing vaccine effect which is considered here takes into account that a fraction of recipients respond to the vaccine and are perfectly protected, whereas the susceptibility of other recipients is unaffected by vaccination; however, it does not recover a “leaky” vaccine effect, under which vaccine responders have incomplete protection. For influenza, vaccine protection is arguably leaky, in that people who exhibit at least a four-fold increase in titer from vaccination, the traditional marker of vaccine immunogenicity and “responder” status, can still be infected. Hence, there is need for improved methods to understand factors influencing differential protection among individuals.

Despite the existing flaws in epidemiological study designs, agreement between lines of experimental evidence and VE estimates supports the hypothesis that immune history is directly related to the vaccine response. Therefore, antigens of influenza strain encountered in childhood are considered to shape the antibody response, thus forming the theory of the “original antigenic sin” (OAS), according to which antibodies to previous, conserved antigens are boosted upon exposure to related strains, often at the expense of responses to novel antigens. The hypothesized mechanisms of low vaccine effectiveness involve complex interactions between infections, vaccinations, competing B- and T-cell populations specific to different epitopes, waxing and waning titers, and age-related changes, among other factors.

Having in mind the existing flaws of epidemiological studies, 2019–2020 reports on influenza VE indicate that, when the vaccine matches the viruses in circulation, the risk is reduced 40% to 60% among the overall population, with better results against influenza B and influenza A (H1N1) viruses than influenza A(H3N2). There is difficulty in determining how effective the influenza vaccine may be; however, studies have shown that the influenza vaccine prevented an estimated 7 million illnesses, 3.7 million medical visits, 109,000 hospitalizations, and 8000 deaths associated with influenza during the 2017–2018 season [7].

In a sum of six studies of VE from September 2019 to January 2020, across 10 European countries in both primary care and hospital settings, in a combined cohort of 31,537 patients with 5310 infections, the VE estimate was 29% to 61% against any influenza in the primary care setting and 35% to 60% in hospitalized older adults (aged 65 years and over) [8]. In a similar interim report from the US from 23 October 2019 to 25 January 2020, in a cohort of 4112 children and adults, the overall VE against any influenza virus associated with medically attended acute respiratory illness was 45%. Interim VE estimates are consistent with those from previous seasons, ranging from 40–60% when influenza vaccines were antigenically matched to circulating viruses [9].

*Reasons for Lack of Response to Influenza Vaccines*

The reason behind partial response to the influenza vaccine leading to “leaky responders” or “non-responders” may include other immune-related factors affecting the ability to kill the virus and also mount antibodies to a given vaccine antigen. This ability should depend in part on HLA genotyping, since, for a successful immune response, a complex between the virus antigen and a suitably matching human leukocyte antigen (HLA) molecule is required. The formation of this complex leads to (a) activation relevant T-cells, which, through HLA Class I, mediate CD8+ cytotoxic T-cells to kill infected cells, and (b) HLA Class II molecules, which mediate CD4+ T-cell activation to initiate the formation of antibodies by B-cells, resulting in adaptive immunity against the virus. Due to selective pressure and co-evolution with pathogens, HLA is the most polymorphic region of the human genome, resulting in substantial individual variability in HLA molecules and thus allowing, in general, for good antigen-HLA Class II molecule matches. However, when such a match fails, e.g., because of lack of appropriate HLA makeup, adaptive immunity is deficient, resulting in chronic disease due to the persistence of offending antigens which could not be eliminated [10]. HLA variations affect antigen binding affinity and consequently contribute to variability in B- and T-cell mediated immune response to pathogens. Furthermore, HLA variation is implicated in treatment of infectious diseases and in vaccine responsiveness [11].

Thus, individual variability in HLA could account for the differences in the magnitude of antibody and T-cell response and the incomplete response observed to all influenza vaccines in otherwise immunocompetent individuals. It appears that there exists a “blind spot” in the efforts to design and develop a universal influenza vaccine, which is created by the lack of consideration of variation in HLA with respect to binding affinity of the different A (H1N1, H3N2) and B strains of influenza. In order to develop effective intervention and durable protection, identification of specific HLA alleles associated with protective immunity is mandated.

The involvement of HLA Class II in the response (VE) to the influenza as well as other vaccines is exemplified by a related report [11] in which thirteen studies (11,686 subjects) evaluated the associations of human leukocyte antigen (HLA) class II and other immunity gene variations with the responses to single vaccines, including influenza virus vaccines, among other. Seven HLA class II genetic variants were included in the meta-analyses. The pooled odd ratios showed that DRB1*07, DQA1*02:01, DQB1*02:01, and DQB1*03:03 were associated with a significant decrease of antibody responses to influenza (and two other) vaccines. On the contrary, DRB1*13 and DRB1*13:01 were associated with a significant increase of antibody responses to the vaccines. Thus, the presence of certain HLA class II alleles may determine the magnitude of antibody responses to influenza vaccination.

Side effects of influenza vaccine have been reported in vivo, and in vitro experiments indicate toxicity of this vaccine leading to compromised cell structure and function, resulting in apoptosis [12]. Vaccine antigens can be harmful to several cell functions; for example, the anthrax vaccine contains a protective antigen that compromises plasma membrane integrity as well as cytoskeletal and mitochondrial function in neuronal cell cultures [13]. Vaccine toxicity often accompanies vaccines using attenuated or inactivated pathogens or their protein components.

*Recent Approaches to Achieve a Better Match of Immunogenic Viral Epitopes with HLA Alleles for Optimal Vaccine Efficacy*

Bioinformatics-based approaches are increasingly gaining traction to aid in the development of epitope-based vaccines. Such in silico approaches have the benefit of avoiding the need to culture pathogenic viruses in vitro. This allows for rapid and economical commercial-scale production of peptide-derived vaccines via bioprocessing pipelines [14]. Web-based databases such as the IEDB (Immune Epitope Database) [15] and the ViPR (Virus Pathogen Resource) [16] repositories offer suites of tools for performing predictions of B- and T-cell antigenicity on peptide sequences. Such predictive tools can be used safely without the requirement of a laboratory, and analyses can be carried out rapidly and repetitively, as reported recently [17].

Thus, the epitope-based in silico approach emerges as powerful and innovative for vaccine manufacturing, which is expanding and promises to yield vaccines in the absence of pre-existing wet lab experiments; it is also deprived of putative side effects of the more conventional previously mentioned vaccines.

This method could be used for developing new vaccines based on specific epitopes of hemagglutinin of the influenza virus, with focus on the protein sequences which are mostly conserved in the different strains and could be protective against many influenza strains. This novel and promising approach would thus contribute towards the formulation of a universal influenza vaccine.

*Conclusions*: The rapidly evolving influenza virus often results in mismatches and low vaccine efficacy in currently existing vaccines, which are mainly egg-based inactivated or live attenuated virus and cell-based vaccines. The shortcomings of existing vaccines discussed above have prompted efforts for next-generation influenza vaccines, which are currently in development. New developing vaccines are based on an array of innovative techniques to shorten production time and increase protection, aiming at a universal influenza vaccine. The main examples of efforts for next generation vaccines aiming at a universal influenza vaccine include: recombinant HA-peptide vaccines (insect and plant-based, among others), DNA and RNA vaccines using different vectors, vaccines with altered glycan composition (monoglycosylated split HA) using recombinant HA, split virus vaccines, newer, live, attenuated influenza vaccines reconstructing the influenza viral genome with synonymous but sub-optimal codons, and epitope-based vaccines, among others.

To conclude the comments on the two reports about influenza vaccines, it is imperative to consider additional innovative methods for vaccine development that yield better results based on antigenic and immunogenic peptide-HLA interactions, utilizing in silico identified HLA epitopes with high affinity for pathogen sequences. Immune mechanisms represent a powerful force, and understanding the ways in which vaccination shapes it by the ability of immune-related receptors to interact with antigens will improve our understanding of vaccine response and vaccine effectiveness. Moreover, it will advance our knowledge on the strengths and the limitations for the development and the use of vaccines against antigenically complex pathogens for which efficacy will be assessed on a background of immune factors, including “immune identity” and immune history, among others.
